# Imbalanced dietary intake alters the colonic microbial profile in growing rats

**DOI:** 10.1371/journal.pone.0253959

**Published:** 2021-06-30

**Authors:** Tae-Hwan Jung, Kyoung-Sik Han

**Affiliations:** 1 Convergence Research Center, Sahmyook University, Seoul, Korea; 2 Department of Food and Nutrition, Sahmyook University, Seoul, Korea; USDA-Agricultural Research Service, UNITED STATES

## Abstract

An imbalanced dietary intake is associated with alteration of intestinal ecosystem. We investigated the impact of imbalanced diets on colonic microbiota, concentrations of short chain fatty acid in colonic digesta and serum immunoglobulins (Igs) of growing rats. Compared to the control diet, consuming diets high in fat, sucrose, or processed meat, or low in iron, increased the abundance of the pathogenic bacteria such as *Clostridium*, *Escherichia coli*, and *Salmonella* species, and decreased the beneficial bacteria, like *Bifidobacteria*, *Lactobacillus*, *Akkermansia*, *Phascolarctobacterium*, *Alistipes*, and butyrate producing species of bacteria in the colon of growing rats. The heatmap of metagenomics indicated that each group was separated into distinct clusters, and the ID group in particular, showed significantly (*P* < 0.01) reduced alpha diversity of colonic microbiota in comparison to the control group. All experimental groups showed significantly (*P* < 0.05 or *P* < 0.01) decreased concentration of acetate and butyrate in the colonic digesta and lower levels of serum IgG or IgA, compared to the control. These results indicated that the imbalanced dietary intake negatively altered intestinal ecosystem and immunity.

## Introduction

The genetic information of gut microbiota is regarded as the second genome of humans. A variety of studies have been conducted on the gut microbiota because its abnormal alteration is closely related with various health disorders such as asthma, obesity, and diabetes [1–3]. Composition of the gut microbiota is influenced by different factors like race, region, and diet [4]. Particularly, dietary intakes may have an exceedingly important effect on the gut microbiota [5], the balance of which may be associated with serum immunoglobulins (Igs) and the fecal short-chain fatty acids (SCFAs) produced by the metabolism of dietary ingredients [6, 7].

The gut is connected to the brain via vagal sensory neurons [8]. The gut microbiota influences the enteric nervous system (ENS), which interacts with the central nervous system (CNS) of brain [9]. The balanced gut microbiota composition contributes to health promotion, whereas its abnormal state can result in the mental disorder by adversely affecting the ENS and CNS [10]. Therefore, the desirable modulation of gut microbiota may prevent, and improve such mental disorders [11].

Recently, it was reported that the gut microbiota composition could affect the mental disorders such as attention deficit hyperactivity disorder (ADHD) and autism [12, 13]. ADHD, a neurodevelopmental disorder, makes it difficult for a person to control impulsive behaviors. It is one of the most common medical conditions in childhood, that tends to persist in adulthood [14]. ADHD is associated with neurotransmitters involved in dopamine function, and also with immune system, which is greatly influenced by alteration in the gut microbiota [15, 16].

Dietary intake has an important role in the modulation of the gut microbiota composition and imbalanced diet leads to dysbiosis of the gut microbiota [5]. Recent studies have reported that aggravation of ADHD symptoms is associated with imbalanced dietary intakes such as high fat (HF), high sucrose (HS), iron deficiency (ID) or processed meat (PM) diet [17, 18]. Prior to study on correlation between dietary patterns and ADHD in human, we have undertaken the present study, designed to investigate the effect of dietary imbalance on the colonic microbiota, production of SCFAs in the colon and serum Igs in growing rats.

## Materials and methods

### Animal study

This work was approved by the Sahmyook University Animal Ethics Committee (SYUIACUC2017-002). The animal procedures were conducted in strict accordance with the National Research Council and Institutional Animal Care and Use Committee (Seoul, Korea).

This study was carried out in the animal facility of Sahmyook University (Seoul, Korea) and all efforts were made to minimize suffering of animals. Experimental animals and feed were purchased from Duyeol Biotech (Seoul, Korea). Sixty Sprague-Dawley male growing rats (140–160 g body weight) were housed singly in stainless steel cages in a room maintained at 22 ± 2°C with a 12 h light-dark cycle. The rats were given one week to acclimatize, during which time they consumed the basal diet and water ad libitum. After adaptation, the rats were randomly allocated to one of the 5 diets (n = 12) during the four weeks of the study (Table 1). The standard diet AIN-93G (Envigo, Indianapolis, IN, USA) was used as a control diet. The HF diet was prepared with adding lard to the control diet. The corn starch was eliminated to increase composition of sucrose in the HS diet. The ID diet was similar to that of control diet, but ferric citrate was excluded from mineral mix. The composition of PM diet was same as that of control diet, but additionally spam (10 g/kg, Austin, MN, USA) was supplied daily. On the last day of the experiment, the rats were quickly anesthetized with carbon dioxide to alleviate pain and sacrified by dislocating the cervical vertebrae. The serum and the colonic digesta of each rat were carefully collected to analyze the composition of gut microbiota, SCFAs, and Igs.

**Table 1:** Composition of the control and experimental diets.

	Diet^a^				
Composition	Control	HF	HS	ID^b^	PM^c^
Ingredient			g/kg diet		
Casein	200	200	200	200	200
Corn starch	397.486	277.486	0	397.698	397.486
Sucrose	100	100	497.486	100	100
Maltodextrin	132	132	132	132	132
Cellulose	50	50	50	50	50
Soybean oil	70	70	70	70	70
Lard	0	170	0	0	0
Vitamin mix	10	10	10	10	10
Mineral mix^d^	35	35	35	34.788	35
L-Cystine	3	3	3	3	3
Choline bitartrate	2.5	2.5	2.5	2.5	2.5
t-Butylhydroquinone	0.014	0.014	0.014	0.014	0.014

^a^ Diet: HF, high fat; HS, high sucrose; ID, iron deficiency; PM, processed meat.

^b^ In the ID group, ferric citrate was eliminated from the mineral mix.

^c^ PM group was added daily the Spam (10 g/kg).

^d^ Mineral mix composition (g/kg): calcium carbonate, 357.0; potassium phosphate, 196.0; potassium citrate, 70.78; sodium chloride, 74.0, potassium sulfate 46.6; magnesium oxide, 24.3; ferric citrate, 6.06; zinc carbonate, 1.65; manganous carbonate, 0.63; cupric carbonate, 0.31; potassium iodate, 0.01; sodium selenate 0.0103; ammonium paramolybdate, 0.008; sodium metasilicate, 1.45; chromium potassium sulfate, 0.275; lithium chloride, 0.0174; boric acid, 0.0815; sodium fluoride, 0.0635; nickel carbonate hydroxide, 0.0318; ammonium metavanadate, 0.0066; sucrose fine ground, 220.7159.

### DNA extraction and quantitative real-time PCR

Bacterial DNA was extracted from the colonic digesta using a QIAamp DNA stool mini kit (Qiagen, Hilden, Germany) according to the manufacturer’s guidelines. Quantitative real-time polymerase chain reaction (qRT-PCR) was performed using the DT-prime 5 (DNA-Technology, Moscow, Russia) with the power SYBR Green PCR master mix (Applied Biosystems, Foster City, CA, USA) and specific primers to measure the number of total bacteria (forward, GCA GGC CTA ACA CAT GCA AGT C; reverse, CTG CCT CCC GTA GGA GT), *Bacteroides* (forward, GAA GGT CCC CCA CAT TG; reverse, CAA TCG GAG TTC GTG), *Lactobacillus* (forward, CGA TGA GTG CTA GGT GTT GGA; reverse, CAA GAT GTC AAG ACC TGG TAA G), *Bifidobacteria* (forward, CTC CTG GAA ACG GGT GG; reverse, GGT GTT CTT CCC GAT ATC TAC A), *Clostridium* (forward, ATG CAA GTC GAG CGA KG; reverse, TAT GCG GTA TTA ATC TYC CTT T), *Enterobacteria* (forward, ATG GCT GTC AGC TCG T; reverse, CTA CTT TTG CAA CCC ACT C), and *Escherichia coli* (forward, GTT AAT ACC TTT GCT CAT TGA; reverse, ACC AGG GTA TCT AAT CCT GTT) based on the methods of Han et al. [19]. The reaction mixture consisted of 10 μL SYBR green master mix, 1 μL each of specific primers (10 pM) and 2 μL DNA sample in a final volume of 20 μL. An initial DNA denaturation step at 95°C for 10 min was followed by 40 cycles of amplification (denaturation at 95°C for 15 s, primer annealing at 55–61°C for 25 s, and extension at 72°C for 30–40 s) and cooling at 4°C. The calibration curve was constituted by Ct values, depending on serial dilutions of bacterial DNA isolated from each variety of bacteria using the DNeasy Blood & Tissue Kit (Qiagen, Hilden, Germany).

### PCR-DGGE

The PCR-DGGE was conducted using the method described by Jung et al. [20]. Extracted bacterial DNA was amplified by PCR using the Veriti 96 well thermal cycler (Applied Biosystems, Foster city, USA), TaKaRa Ex Taq polymerase (Takara, Kusatsu, Japan), and primers (forward, CGC CCG GGG CGC GCC CCG GGC GGG GCG GGG GCA CGG GGG GAA CGC GAA CCT TAC; reverse, CGG TGT GTA CAA GAC CC). The PCR amplicons were subjected to PCR-denaturing gradient gel electrophoresis (DGGE) using the Dcode system (Biorad, Hurcules, CA, USA). A polyacrylamide gel (8%) containing the urea-formamide gradient of 35–50% was used for the separation of the PCR amplicons, and the electrophoresis was conducted at 130 V for 5 h at 60°C. The intensity of each band was measured using the Image Lab 5.1 Software (Biorad) and sixteen bands of specific interest, indicating different intensities among the groups were resected. The DNA from each band was cloned separately into the pGEM-T Easy Vector (Promega, Madison, WI, USA), and sequenced at Bionics company (Seoul, Korea). Nucleotide sequence data was analyzed using the GenBank database with the BLAST search program.

### Metagenomics analysis

Metagenomic analysis for gut microbiota was conducted at Chunlab company (Seoul, Korea). PCR amplification was performed using fusion primers targeting from V3 to V4 regions of the 16S rRNA gene in the extracted DNA. For bacterial amplification, fusion primers of 341F (5’-TGATACGGCGACCACCGAGATCTACAC-XXXXXXXXTCGTCGGCAGCG TCAGATGTGTATAAGAGACAG-CCTACGGGNGGCWGCAG-3’; underlining sequence indicates the target region primer) and 805R (5’- CAAGCAGAAGACGGCATACGAGAT-XXXXXXXX-GTCTCGTGGGCTCGG-AGATGTGTATAAGAGACAG-ACTACHVGGGTATCTAATCC-3’). The Fusion primers are constructed in the following order which is P5 graft binding, i5 index, nextera consensus, sequencing adaptor, and target region sequence. The amplifications were carried out under the following conditions: initial denaturation at 95°C for 3 min, followed by 25 cycles of denaturation at 95°C for 30 sec, primer annealing at 55°C for 30 s, and extension at 72°C for 30 s, with a final elongation at 72°C for 5 min. The PCR product was confirmed by using 1% agarose gel electrophoresis and visualized under a Gel Doc system (BioRad). The amplified products were purified with the CleanPCR (CleanNA, Waddinxveen, Netherlands). Equal concentrations of purified products were pooled together, from which short fragments (non-target products) were removed with CleanPCR (CleanNA). The quality and product size were assessed on a Bioanalyzer 2100 (Agilent, Palo Alto, CA, USA) using a DNA 7500 chip. Mixed amplicons were pooled and the sequencing was carried out at Chunlab, Inc. (Seoul, Korea), with Illumina MiSeq Sequencing system (Illumina, Sandiego, CA, USA) according to the manufacturer’s instructions. The data analysis was performed using CLcommunity^TM^ ver 3.46 software (Chunlab) and EZBioCloud database.

### Measurement of SCFA

The SCFAs (acetate, propionate, butyrate) were measured using gas chromatography. Ten milligrams of the dried colonic digesta were mixed with 1 mL of the methanol, and shaken at 200 rpm for 90 mins at 25°C. After the mixture was centrifuged at 10,000 rpm for 10 min at 25°C, the supernatants were filtered through a 0.22 μm syringe filter, and 2 μL of the filtrates were injected into YL 6100 GC column (30 m x 0.25 mm, Youngin Chromass, Gyeonggido, Korea) to analyze SCFAs from the colonic digesta. The calibration curves were plotted using the standard reagents (Sigma-Aldrich, Saint Louis, MO, USA).

### Measurement of immunoglobulins

Concentration of immunoglobulins (IgG, IgM, and IgA) in the serum were measured using the Rat IgG total uncoated ELISA kit (Invitrogen, Carlsbad, CA, Austria), Rat IgM ELISA Kit (Bethyl, Montgomery, TX, USA), and Rat IgA ELISA kit (Bethyl) according to the manufacturer’s guidelines using coating and detection antibodies at room temperature. The absorbance values were measured at 450 nm using the microplate reader (Emax, Molecular Devices, San Jose, CA, USA).

### Statistical analysis

The results were presented as mean ± SEM. Significance of the differences among the groups was determined using X^2^ test and the SAS/PROC GLM software (SAS version 9.1; SAS Institute Inc., Cary, NC, USA). The two-tailed p value less than 0.05 were considered significant.

## Results

### Measurement of colonic microbiota by qRT-PCR

The qRT-PCR was conducted to estimate the number of total bacteria, *Bacteroides*, *Lactobacillus*, *Bifidobacteria*, *Clostridium*, *Enterobacteria*, and *E*. *coli* strains. As shown in Table 2, there was no significant difference between control and experimental groups on the number of total bacteria, *Bacteroides* group, and *Lactobacillus* strains. However, each experimental group showed considerable difference in abundance of *Bifidobacteria*, *Clostridium*, *Enterobacteria*, and *E*. *coli* strains in comparison to the control group. The number of *Clostridium*, *Enterobacteria*, and *E*. *coli* strains significantly (*P* < 0.05 or *P* < 0.01) increased in rats fed HF, ID, or PM diet compared to the control group, whereas those fed HS diet showed a significantly (*P* < 0.01) lower number of *Bifidobacteria* in comparison to the control group. A significantly (*P* < 0.05 or *P* < 0.01) lower ratio of *Lactobacillus* to *Enterobacteria* was also observed in animals on the ID or PM diet compared to the control.

**Table 2:** The counts (log_10_ 16S rDNA gene copies g^-1^ of colonic digesta) of different bacteria groups measured by quantitative real-time PCR.

	Diet^a^				
Bacteria	Control	HF	HS	ID	PM
Total bacteria	10.47±0.11^b^	10.50±0.07	10.51±0.08	10.63±0.13	10.26±0.15
*Bacteroides* group	9.13±0.09	9.15±0.10	9.22±0.12	9.00±0.23	8.77±0.23
*Lactobacillus*	9.09±0.20	9.24±0.23	8.84±0.12	9.32±0.17	8.71±0.17
*Bifidobacteria*	8.59±0.15	8.66±0.26	7.59±0.21**^c^	8.54±0.16	8.35±0.27
*Clostridium*	6.40±0.23	8.07±0.47**	6.82±0.27	8.21±0.54**	7.89±0.59*
*Enterobacteria*	6.56±0.18	6.91±0.16	6.30±0.16	8.01±0.20**	6.95±0.12
*Escherichia coli*	6.63±0.21	6.79±0.16	6.17±0.13	7.92±0.22**	6.90±0.14*
*Lactobacillus*: *Enterobacteria*	2.56±0.27	2.33±0.26	2.54±0.15	1.31±0.21**	1.76±0.18*

^a^ Diet: HF, high fat; HS, high sucrose; ID, iron deficiency; PM, processed meat.

^b^ Values are means ± SEM, n = 12.

^c^ Statistical significance compared to control was accepted at

*P < 0.05 or

**P < 0.01.

### PCR-DGGE and DNA sequencing

**Fig 1:**
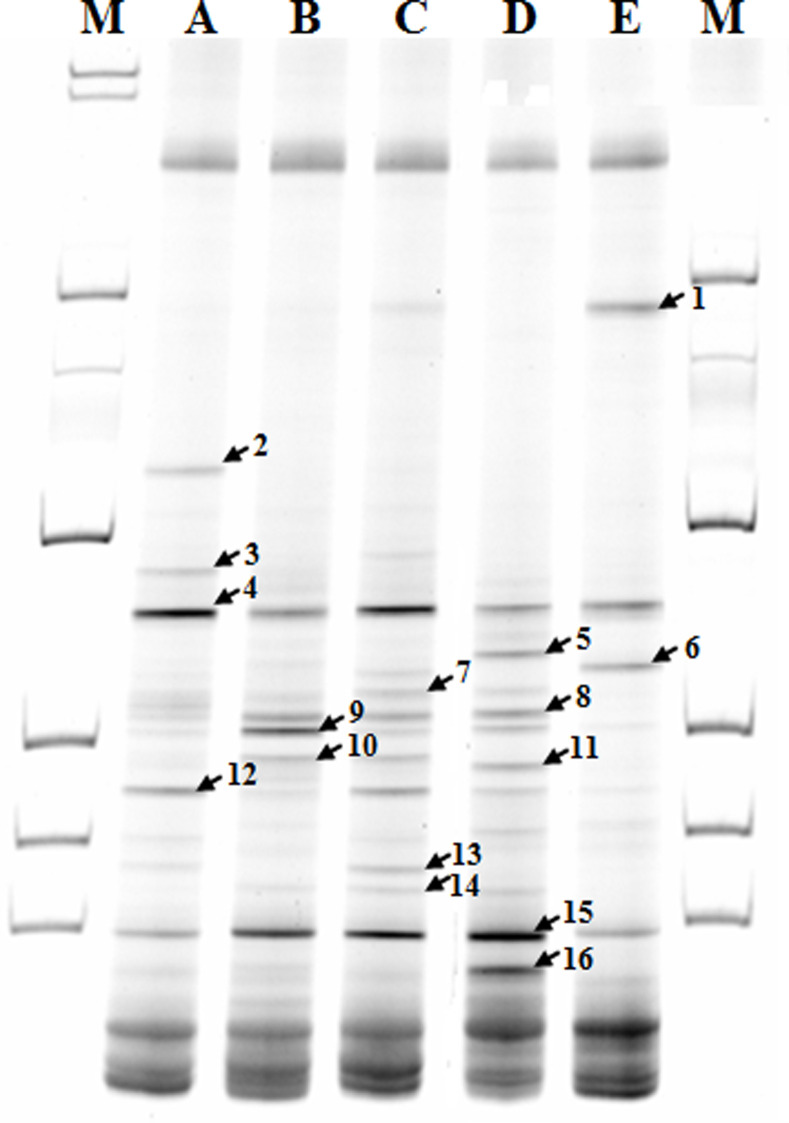
PCR-DGGE bands from pooled DNA samples (n = 12) of colonic digesta used in DNA sequencing for identification of bacteria. Arrows indicate the bands subject to DNA sequencing. M, marker; A, control; B, high fat; C, high sucrose; D, iron deficiency; E, processed meat.

The DGGE band pattern of pooled samples of each group (n = 12) is shown in Fig 1. The rats maintained on the experimental diets showed distinct band patterns compared to the control. The DNA sequencing of 16 selected DGGE bands revealed that the intensity of bands (No. 2 and 3) corresponding to beneficial bacteria such as *Akkermansia muciniphila and* butyrate-producing bacterial strains in the experimental groups decreased compared to the control; and that of bands (No. 4 and 12) representing *Lactobacillus* strains was reduced in the experimental groups except the rats fed the HS diet when compared to the control group, whereas that of bands (No. 5, 7, 8, 9, 15, and 16) representing the pathogenic bacteria such as *E*. *coli*, *Shigella*, *Salmonella*, and *Clostridium* increased in rats fed the experimental diets in comparison to those on control diet (Table 3). In particular, the ID group showed that the most pronounced increase in the intensity of bands corresponding to harmful bacteria such as *E*. *coli*, *Salmonella*, and *Clostridium* strains increased compared to the control group.

**Table 3:** Bacterial species identified using DNA sequencing of the PCR-DGGE bands.

Band No.	Description	Similarity with nearest neighbor, %	Accession No.
1	Proteobacterium symbiont of Nilaparvata lugens clone TM85-7	97–99	FJ774967.1
2	*Akkermansia muciniphila* strain GP06	99–99	KT340084.1
3	Butyrate-producing bacterium PH07AY02	99–99	DQ144124.1
4	*Lactobacillus johnsonii* strain LJ	99–99	KY751911.1
5	*Escherichia coli* strain GE3	99–99	CP012376.1
6	Enterobacteriaceae bacterium 351UGen	99–99	GQ152407.1
7	*Shigella sonnei* strain 2015C-3566	99–99	CP022457.1
8	*Salmonella enterica* subsp. *enterica* serovar Typhi isolate E98_3139-sc-1927833 genome assembly	100–99	LT905143.1
9	*Salmonella enterica* subsp. *enterica* strain 08–00436	99–99	CP020492.1
10	*Bacteroides dorei* CL03T12C01	99–99	CP011531.1
11	Uncultured Blautia sp. clone ClosJI82708_82B07_528	99–99	JX229024.1
12	*Lactobacillus* sp. ST-465-5-N	99–99	KR364778.1
13	Uncultured bacterium clone CFT19A8	99–94	DQ455829.1
14	Dorea longicatena gene, strain: JCM 11232	99–99	LC037228.1
15	*Clostridium* sp. CS1 gene	98–99	AB586147.1
16	*Escherichia coli* strain WAT	99–99	CP012380.1

### Metagenomic analysis

**Fig 2:**
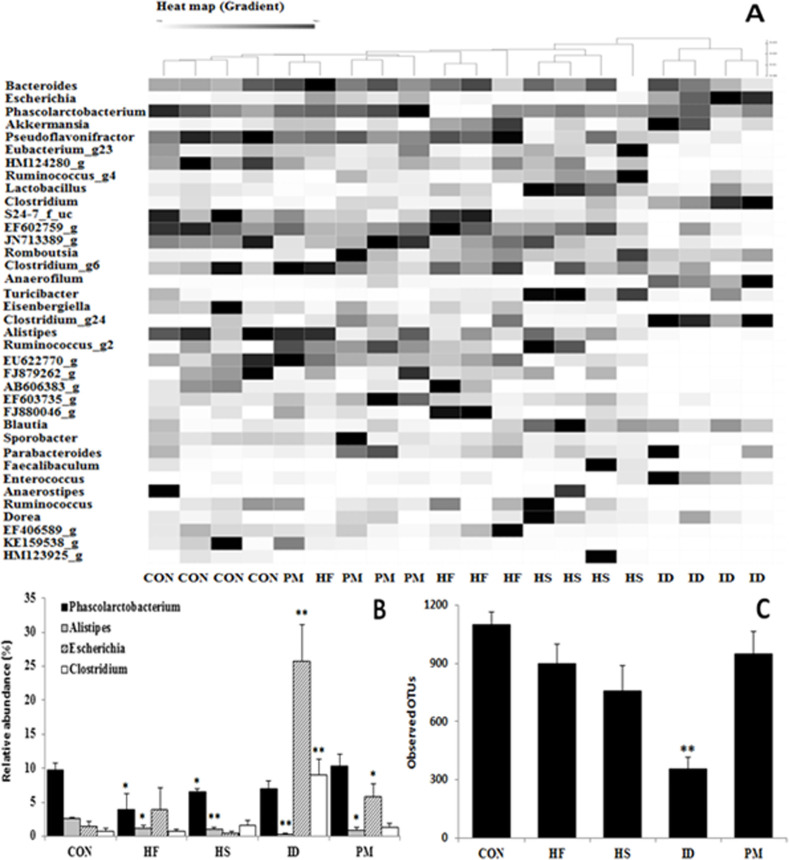
Comparison of the gut microbiota at genus level between control and experimental groups. (A) heatmap, (B) relative abundance, (C) alpha diversity. CON, control; HF, high fat; HS, high sucrose; ID, iron deficiency; PM, processed meat. Values are expressed as mean ± SEM. Statistical significance compared to control was accepted at *P < 0.05, **P < 0.01.

Metagenomic analysis was conducted to investigate the composition and diversity of microbiota in the colonic digesta of rats from experimental and control groups. Processing raw reads started with quality check and filtering of low quality (<Q25) reads by Trimmomatic 0.32 [21]. The paired-end sequence data were merged using fastq_mergepairs command of VSEARCH 2.13.4 [22] with default parameters. Primer sequences were then trimmed with ChunLab’s in-house program at a similarity cut off of 0.8. Non-specific amplicons that did not encode 16S rRNA were detected by using HMMER program with hmm profiles [23]. Unique reads were extracted and redundant reads were clustered with the unique reads by derep_fulllength command of VSEARCH. The EzBioCloud database was used for taxonomic assignment using usearch_global command of VSEARCH followed by more precise pairwise alignment [24]. Chimeric reads were filtered on reads with <97% similarity by reference based chimeric detection using UCHIME [25] algorithm6 and the non-chimeric EzBioCloud database. The sequence data were then clustered using CD-HIT [26], UCLUST [27], and defined OTUs in this step. The heatmap was generated based on strains containing at least 1% at the genus level using CLcommunity^TM^ software. The heatmap analysis of different genera of the colonic microbiota showed that the microbiota profile of the control and experimental groups mostly belonged to distinct clusters (Fig 2A). The colonic microbiota profiles at genus level from control and experimental groups revealed that the proportion of harmful bacteria such as *Escherichia* or *Clostridium* significantly (*P* < 0.05) increased in the ID and PM groups, whereas the ratio of *Phascolarctobacterium* or *Alistipes* strains, which induce SCFA production, markedly (*P* < 0.05) decreased in rats fed with experimental diets, as compared to those on control diet (Fig 2B). In addition, we analyzed the alpha diversity using the number of observed OTUs. There was no significant difference in alpha diversity of colonic microbiota among the test groups except that the rats fed ID diet showed significantly (*P* < 0.01) decreased alpha diversity in comparison to the control group (Fig 2C).

### Measurement of SCFAs and immunoglobulins

**Fig 3:**
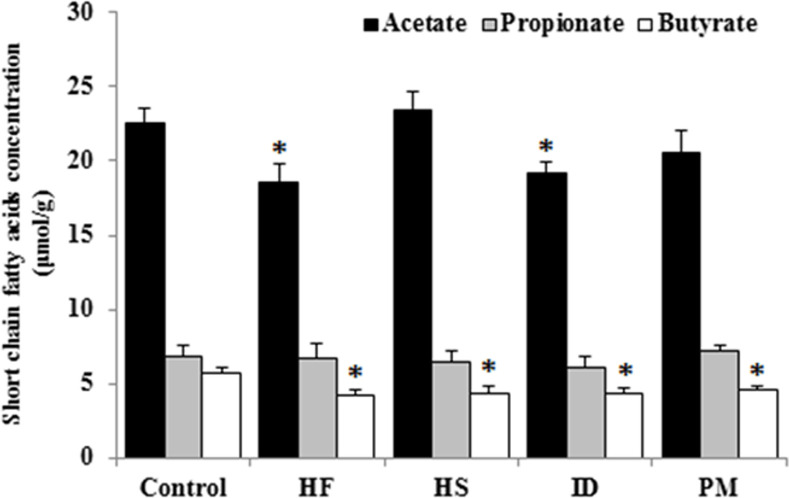
Concentration of short chain fatty acids in the colonic digesta according to dietary treatments. HF, high fat; HS, high sucrose; ID, iron deficiency; PM, processed meat. Values are expressed as mean ± SEM. Statistical significance compared to control was accepted at *P < 0.05.

The SCFAs (acetate, propionate, and butyrate) in the colonic digesta were measured using gas chromatography. As shown in Fig 3, there was significant (*P* < 0.05) decrease in the acetate or butyrate among the experimental groups, compared to the control. HF and ID groups showed significant (*P* < 0.05) decrease in the acetate concentration, while animals in all experimental diet groups showed significantly (*P* < 0.05) lower concentration of butyrate, than those of the control group.

**Fig 4:**
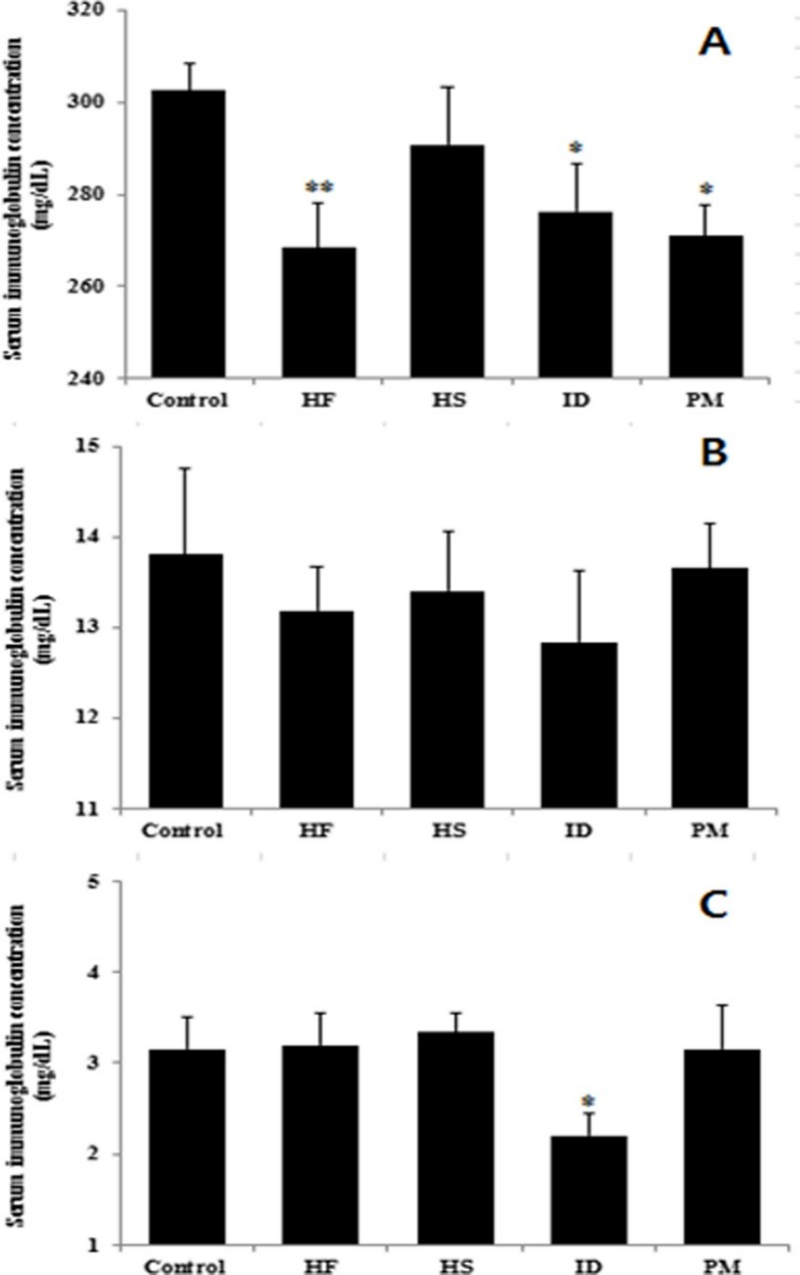
Immunoglobulin levels in the serum of growing rats with different dietary treatments. (A) IgG, (B) IgM, (C) IgA. HF, high fat; HS, high sucrose; ID, iron deficiency; PM, processed meat. Values are expressed as mean ± SEM. Statistical significance compared to control was accepted at *P < 0.05, **P < 0.01.

The concentration of the IgG, IgM, and IgA in the serum was estimated using the ELISA kits. As shown in Fig 4, there was no statistical difference in IgM concentration of the serum between control group and experimental groups. However, The IgG concentration in the serum significantly (*P* < 0.05) decreased in the rats fed HF, ID or PM diets compared to the control group. Moreover, IgA concentration in the serum of ID group significantly (*P* < 0.05) reduced in comparison to the control group.

## Discussion

Gut microbiota plays an important role in maintaining health by regulating the metabolites such as SCFAs and molecules like immunoglobulins, and protect the host from external pathogens and potentially harmful bacteria residing in the intestine [28].

Despite limitation of PCR-DGGE method that different species with similar sequences might migrate to the same position on DGGE gel, the method has been used to study the taxonimic of bacterial community in the rats. In addition, the qRT-PCR method can be used to investigate alteration of the abundance of particular species. The results of the qRT-PCR and PCR-DGGE indicated that the rats fed HF, HS, ID, or PM diet had increased abundance of the pathogenic bacteria such as *Clostridium*, *E*. *coli*, and *Salmonella* strains, and reduced abundance of the beneficial bacteria such as *Bifidobacteria*, *Lactobacillus*, *Akkermansia* or butyrate producing bacterial strains when compared to the control. The decrease in abundance of *Bifidobacteria* strains in the rats fed HS diet seems to be due to the low in corn starch compared to control diet. In addition, it was observed that the ratio of *Lactobacillus* to *Enterobacteria* was markedly reduced in the rats fed ID or PM diet in comparison to the control. This ratio is known as an index of healthy intestine and the high index indicates greater intestinal protection against opportunistic pathogens [29].

The beneficial bacterial strains, such as *Lactobacillus* and *Bifidobacteria* modulate the balance of gut microbiota composition, interfering with the toxicity of pathogenic strains, and may have beneficial effects on psychological health [30]. *Akkermansia* strains improve health by increasing the thickness of mucus and intestinal barrier function [31, 32]. Abundance of these bacteria usually lead to a relative decrease in the pathogenic strains of bacteria, like *Salmonella*, *Staphylococcus*, *E*. *coli* and *Clostridium* [33]. Furthermore, a variety of studies announced that the higher abundance of *Clostridium* and *E*. *coli* was observed in the children with psychological problems compared to healthy children [34, 35].

The heatmap analysis indicated the existence of five distinct clusters, which meant that each group had distinct profiles of gut microbiota. The relative abundance of the pathogenic bacteria such as *Escherichia* and *Clostridium* strains significantly increased in rats fed ID or PM diets compared to the control, and these observations match with the results of qRT-PCR and PCR-DGGE. It was also observed that in all experimental groups, the relative abundance of the *Phascolarctobacterium* or *Alistipes* strains significantly decreased in comparison to that in the control group [36]. These bacteria are closely associated with the production of butyrate, which is important for maintaining intestinal health by promoting a favorable environment for the growth of beneficial bacteria [37]. Alpha diversity of colonic digesta significantly decreased in the rats fed ID diet in comparison to that of the control. Pereira et al. [38] reported that growing rats fed iron-deficient diet were shown to have markedly lower microbial diversity in the feces than rats fed iron-sufficient diet; and this decrease was partially restored with optimum supplementation of iron. Moreover, iron deficiency induces increased fecal calprotectin level and inflammation of the intestine, resulting in the increase in pathogens [39].

In this study, there was no significant difference in the propionate concentration in colonic digesta, but rats fed HF or ID diet showed considerably decreased fecal concentrations of acetate and butyrate, when compared to the control. The SCFAs, such as acetate and butyrate, can regulate the intestinal homeostasis and have direct effects on the composition of gut microbiota [6]. It is reported that SCFAs inhibit the growth of pathogenic bacterial strains, such as *Salmonella*, *E*. *coli*, and *Clostridium* [40]. In addition, the butyrate has an important role in modulating the neuronal excitability, and the activity of ENS [41]. Dalile et al. [42] reported that the SCFAs are known to play a key role in the microbiota-gut-brain axis of interaction, although the definite mechanism and the pathway are not yet identified.

The serum IgM did not statistically differ among the experimental groups but the serum IgG and IgA significantly decreased in rat fed HF, ID, or PM diet compared to that in the control. IgG promotes the growth of beneficial bacteria, inhibiting that of the pathogens in the intestine, and IgA is also important in modulating the composition of gut microbiota and immune function [43]. Al-Saiady [44] found that increased concentration of serum IgG was associated with relative abundance of the beneficial bacteria such as *Lactobacillus* strains.

## Conclusions

In the present study, we investigated the influence of imbalanced diets on the gut microbiota, and levels of SCFAs in the colonic digesta and serum immunoglobulins through animal experiment. The results indicated that imbalanced dietary intake, especially ID diet, induced dysbiosis of the gut microbiota, and reduction of fecal SCFAs and serum Igs. Although the result of this study has a limitation which is not accompanied by human trial, the imbalanced dietary intake negatively altered intestinal ecosystem and immunity.

## Supporting information

S1 Raw images PCR-DGGE bands from pooled DNA samples (n = 12) of colonic digesta used in DNA sequencing for identification of bacteria. M, marker; A, control; B, high fat; C, high sucrose; D, iron deficiency; E, processed meat.
(PDF)
