# Supplementary figures and images for: Imbalanced dietary intake alters the colonic microbial profile in growing rats
Source: PLoS One. 2021 Jun 30;16(6):e0253959. doi: 10.1371/journal.pone.0253959 (PMC8244856; doi:10.1371/journal.pone.0253959)

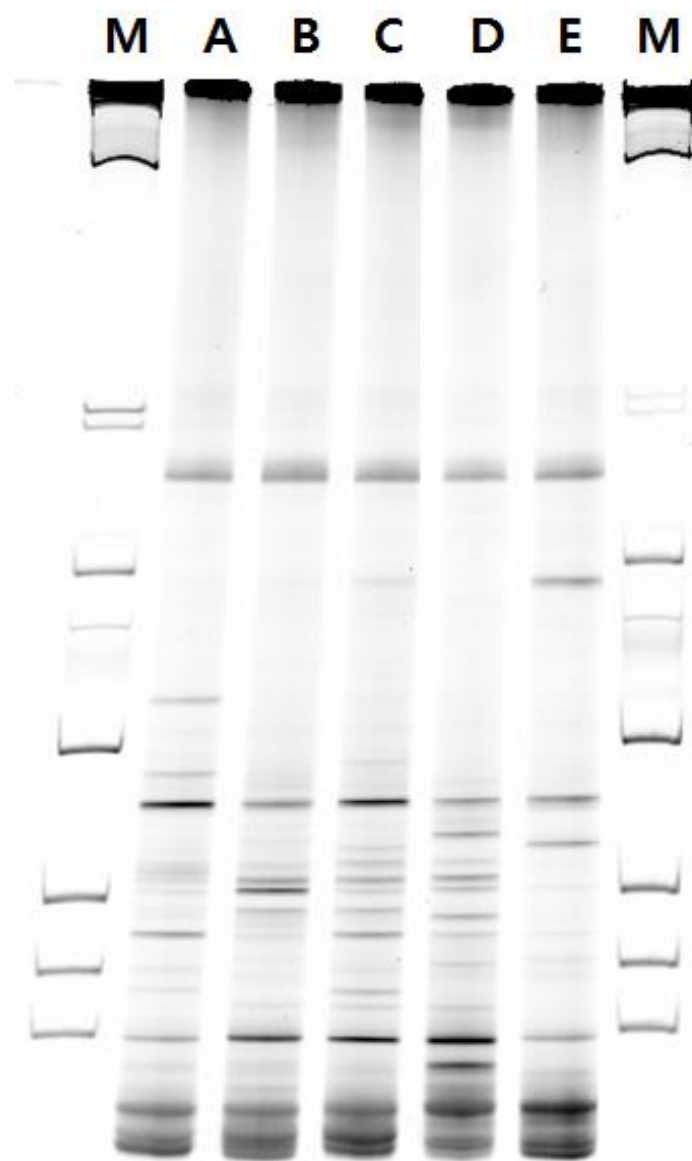

Supplement: S1 Raw images — M, marker; A, control; B, high fat; C, high sucrose; D, iron deficiency; E, processed meat. (PDF) [file pone.0253959.s001.pdf]
